# Mechanism of Acrosome Biogenesis in Mammals

**DOI:** 10.3389/fcell.2019.00195

**Published:** 2019-09-18

**Authors:** Muhammad Babar Khawar, Hui Gao, Wei Li

**Affiliations:** ^1^State Key Laboratory of Stem Cell and Reproductive Biology, Institute of Zoology, Chinese Academy of Sciences, Beijing, China; ^2^University of Chinese Academy of Sciences, Beijing, China

**Keywords:** acrosome biogenesis, autolysosome, lysosomes, globozoospermia, spermiogenesis

## Abstract

During sexual reproduction, two haploid gametes fuse to form the zygote, and the acrosome is essential to this fusion process (fertilization) in animals. The acrosome is a special kind of organelle with a cap-like structure that covers the anterior portion of the head of the spermatozoon. The acrosome is derived from the Golgi apparatus and contains digestive enzymes. With the progress of our understanding of acrosome biogenesis, a number of models have been proposed to address the origin of the acrosome. The acrosome has been regarded as a lysosome-related organelle, and it has been proposed to have originated from the lysosome or the autolysosome. Our review will provide a brief historical overview and highlight recent findings on acrosome biogenesis in mammals.

## Introduction

Sexual reproduction requires the fusion of two gametes in a critical multistep process termed fertilization. To ensure the success of fertilization, each step needs to proceed in a very precise manner. One of the key steps that ensures successful fertilization is acrosome reaction (AR). Sperm-egg fusion is a carbohydrate-dependent event that takes place via interaction between several glycan-binding molecules (receptors) present on the sperm plasma membrane with their corresponding glycans (ligands) localized to the zona pellucida (oocyte) (Yanagimachi, 1994; Tulsiani et al., 1997; Shur, 1998; Töpfer-Petersen, 1999; Wassarman, 1999). This irreversible interaction of the gametes leads to a calcium-mediated signal transduction cascade of events that results in the release of the acrosomal contents via exocytosis, which is termed AR. Several hydrolytic and proteolytic acrosomal enzymes are released in order to facilitate sperm fusion with the oocyte (Ikawa et al., 2010). Any structural or functional acrosomal abnormality could impair sperm fusion, and ultimately result in infertility. Moreover, studies have shown that intra-cytoplasmic insemination with sperm containing acrosomal abnormalities did not lead to successful fertilization, even in the absence of fertilization barriers, because the oocyte was unable to be efficiently activated (Nasr-Esfahani et al., 2008, 2010a,b). Only intracytoplasmic sperm injection (ICSI) followed by assisted oocyte activation with calcium ionophore was found to achieve high live birth-rates (Shang et al., 2019). Thus, the acrosome is indispensable for fertilization.

Acrosome biogenesis in mammals is accompanied with spermatid differentiation during spermiogenesis, which is characterized by the transformation of a spermatid into a spermatozoon. The process continues throughout the reproductive lifespan of the male. Although the acrosome morphology varies from species to species, two basic parts make up the acrosome in all mammals: (1) a large anterior part that varies in shape (paddle, hook, and spatula-like) and in size (Bedford, 2014) and (2) an equatorial segment (ES), which is the smaller and thinner part of the acrosome found in the middle of the sperm head. The acrosomal contents are enclosed in a single membrane that is generally divided into an outer acrosomal membrane (OAM) and an inner acrosomal membrane (IAM). The OAM lies immediately beneath the plasma membrane of the spermatid and both of these membranes fuse at the time of AR (Yanagimachi, 2011). The IAM lies above the nuclear envelope as a cap and does not fuse during AR. The luminal contents are heterogeneous and are usually categorized as soluble and particulate material. The soluble material is comprised of hydrolytic enzymes that take part in AR and help to disperse the oocyte coverings. The particulate material is the acrosomal matrix that facilitates the sperm-oocyte interaction during fertilization by providing a stable protein scaffold (Buffone et al., 2004; Suryavathi et al., 2015).

**Figure 1:**
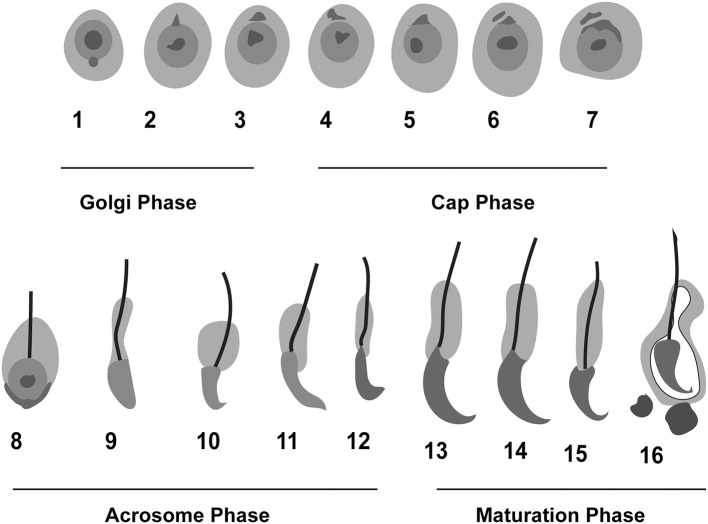
Schematic representation of the phases of acrosome biogenesis in mouse. Round spermatids are transformed into mature spermatozoa in four different phases: Golgi (1–3), Cap (4–7), Acrosome (8–12), and maturation phase (13–16). The whole process of spermiogenesis is comprised of 16 steps in mouse.

Researchers have characterized the process of spermiogenesis in mice, and they have identified 16 steps, with acrosome biogenesis being a key event. Acrosome biogenesis is classically divided into four major phases: Golgi (1–3 steps), cap (4–7 steps), acrosome (elongation) (8–12 steps), and maturation (final) phases (13–16 steps) (Figure 1). This four-phase division of acrosome biogenesis was proposed more than half-a-century ago, based mainly on light microscopic analysis of testicular sections stained with periodic acid–Schiff (PAS) (Clermont and Leblond, 1955).

**Figure 2:**
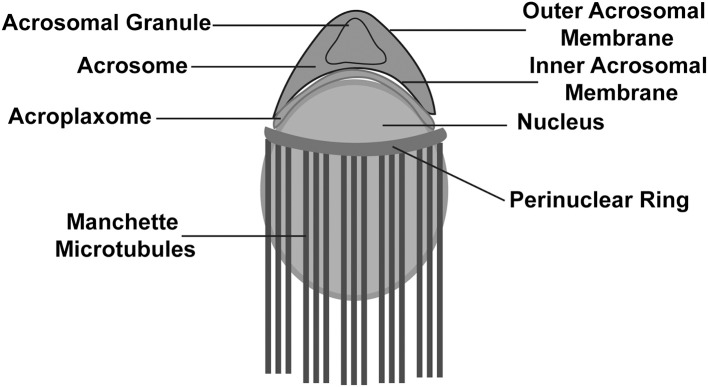
Schematic illustration of a developing spermatid. Developing spermatid has an elongating nucleus surrounded by a perinuclear ring and manchette microtubules. The acrosome lies above the nucleus and is attached to the nucleus with assistance from the acroplaxome.

The 1st phase is called the Golgi phase because the Golgi apparatus is an essential organelle that supports early spermiogenesis (Leblond and Clermont, 1952; Hess, 1990; Russell et al., 1993). During the 1st phase, the Golgi apparatus is very active in producing several glycoproteins, and the trans-Golgi network gives rise to several small proacrosomal vesicles that are required for the formation of a mature acrosome. These proacrosomal granules fuse to form a large solitary acrosomal granule near the concave region of the nuclear surface. The central part of the acrosomal granule is bound to the nuclear envelope while the peripheral part is associated with the perinuclear theca (BOX 1; Figure 2). In the 2nd (Cap) phase, the acrosomal granule becomes enlarged with glycoprotein-rich contents. Moreover, it begins to flatten upon touching the nuclear envelope, and spreads over the nucleus to form a cap. At the same time, the Golgi complex moves to the nascent neck region located in the distal end. The acrosomal granule gradually covers 1/3 of the nuclear surface and spreads, transforming into a very thin layer (Figure 1). Near the distal end of the developing acrosome, the “acroplaxome” can be found, which is an important structure that supports spermiogenesis. It consists of a marginal ring, which consists of an acrosomal plate that is made up of keratin and F-actin (BOX 1; Figure 2). At the time of elongation of the spermatid head, the marginal ring is associated with the growing edge of the acrosome and the nuclear surface (nuclear plate). Thus, acroplaxome not only binds the acrosome with the nucleus but also ensures the developing acrosomal cap remains anchored to the nuclear envelope undergoing elongation (Kierszenbaum et al., 2003). In the 3rd (acrosomal) phase, the acrosomal system begins to migrate over the ventral surface of the elongating spermatid nucleus and this migration ends in step 14 spermatid (BOX 1; Figure 1; Hess and de Franca, 2008). At this stage, the acrosome undergoes condensation and attaches itself to the IAM, while chromatin also undergoes intense condensation. Several cytoskeletal proteins including calmodulin (Camatini et al., 1992), actin (Talbot and Kleve, 1978), and α-spectrin-like antigens (Virtanen et al., 1984), play very important roles in the acrosomal organization. The elongating spermatids show initiation of manchette microtubule formation near the nuclear ring region (perinuclear ring), thinning of the cytoplasm and a gradual orientation of the acrosome toward the overlying plasma membrane (BOX 1; Figure 2). The 4th (maturation) phase involves a few changes in nuclear morphology and acrosomal migration. Condensation of the nucleus continues and acrosomal granule spreads over the entire acrosomal membrane and the acrosome differentiates into anterior and posterior regions. The anterior portion becomes the acrosome apex, while the rest of the acrosome covers nearly all the nuclear surface, except the part attached to the sperm tail (Russell et al., 1993). Moreover, excess cytoplasm, cytoplasmic components (lipids) and multiple unwanted organelles including mitochondria, vesicles, and ribosomes are disposed of in the form of cytoplasmic droplets prior to spermiation (Figure 1; Hess et al., 1993; Russell et al., 1993; De Franca et al., 1995). The resulting residual bodies released from spermatids are taken up and digested by Sertoli cells. Although, the morphogenetic process of the acrosome is well studied, the precise molecular mechanism of sperm acrosome biogenesis is not yet fully understood. Here, we describe the most recent progress that has been made in understanding the molecular mechanism underlying acrosome biogenesis.

Box 1 **Glossary.**

**Perinuclear theca:** a condensed cytoskeletal structure that encompasses the nucleus of the mammalian spermatozoa, except near the tail implantation region. The structure consists of two distinct regions, a subacrosomal layer/perforatorium, and postacrosomal regions.

**Acroplaxome** (Greek words akros, topmost; platys, flat; soma, body): a structure located in the sub-acrosomal space that holds the developing acrosome to the spermatid nucleus.

**Manchette:** is a temporary structure that persists only during elongation and encompasses the elongating spermatid head. The structure lies below the marginal ring of the acroplaxome and consists of a perinuclear ring and inserted microtubular mantle.

**Step 14 Spermatid:** Spermiogenesis occurs in 16 steps (1-16) in mice. Step 14 spermatids are characterized by chromatin condensation, nucleus elongation, and transformation of the head in sickle a shape.

## The Molecular Mechanism Underlying Acrosome Biogenesis

Several ER and Golgi-associated proteins actively participate in acrosome biogenesis. The endoplasmic reticulum (ER) is the main site of protein synthesis and folding (Vitale et al., 1993), while the Golgi apparatus directs glycosylation, processing, and sorting of newly synthesized proteins by the ER. The biosynthesis of some acrosome-specific proteins, such as acrosin, starts during the meiotic pachytene stage and continues as round spermatids enter the elongation stage (Anakwe and Gerton, 1990; Kashiwabara et al., 1990; Escalier et al., 1991). These proteins then enter the exocytic route and are transported to their respective targeted area in the form of proacrosomal granules that originate from the Golgi apparatus. The presence of these proacrosomal granules in the pachytene stage has been confirmed by numerous studies (Nicander and Ploen, 1969; Fawcett, 1975; Anakwe and Gerton, 1990; Suarezquian et al., 1991; Ramalho-Santos et al., 2002). Moreover, the presence of several ER-associated proteins such as protein O-mannosyltransferase 1 (POMT1) (Prados et al., 2007), POMT2 (Willer et al., 2002) and Calreticulin (Nakamura et al., 1993), have also been detected in the acrosome. Another ER-associated protein that has been detected in mouse testes is HSP90B1 (gp96/Grp94; glucose-related protein 94) (Asquith et al., 2005; Yang and Li, 2005). Germ cell-specific *Hsp90b1* knockout resulted in spermatozoa characterized by globular/abnormal heads, similar to those in globozoospermia syndrome (Audouard and Christians, 2011). Therefore, HSP90B1 has been suggested to be a testis-specific chaperone and to be required for the proper folding of acrosomal proteins. The impairment of protein folding during HSP90B1 deficiency suggests an important role of ER protein folding in acrosome biogenesis. β-Glucosidase 2 (GBA2) is another ER-associated protein, and is involved in the metabolism of bile acid–glucose conjugates (Matern et al., 1997). GBA2 disruption results in the formation of abnormal spermatozoa characterized by enlarged heads and the absence of acrosome (Yildiz et al., 2006). In short, these proteins are indispensable for acrosome biogenesis.

In addition, the highly dynamic trafficking of the Golgi-derived vesicles is also involved in acrosome biogenesis. For instance, several Golgi proteins, including Golgin-95/GM130, Golgin-97, Giantin, and β-COP have been found in acrosomal associated membranes (Moreno and Schatten, 2000; Moreno et al., 2000; Ramalho-Santos et al., 2001; Hermo et al., 2010). Among these proteins, β-COP and Clathrin have been described to participate in anterograde and retrograde transport of vesicles during acrosome formation (Martínez-Menárguez et al., 1996b; Moreno et al., 2000; Ramalho-Santos et al., 2001). Golgi-derived vesicle trafficking during acrosome biogenesis can generally be divided into three steps: vesicle formation, trafficking, and fusion. Some of the important proteins that contribute to these three events are described below.

Vesicle formation and trafficking are key events of acrosome biogenesis. Stromal membrane-associated protein 2 (SMAP2) regulates the production of clathrin-coated vesicles from the trans-Golgi network (TGN) by interacting with CALM (Clathrin and the Clathrin assembly protein) and Syntaxin 2 (a component of SNARE complex that helps in membrane fusion), contributing to acrosome biogenesis (Funaki et al., 2013). GOPC (Golgi-associated PDZ- and coiled-coil motif-containing protein) was identified as a frizzled-interacting protein that is involved in vesicular trafficking from the Golgi apparatus (Yao et al., 2001). GOPC is predominantly localized in the trans-Golgi region in round spermatids and plays an important role in the transport and fusion of the proacrosomal vesicles with the growing acrosome (Yao et al., 2002). Golgin subfamily A member 3 (GOLGA3) is another Golgi-associated protein that is highly expressed during the round spermatid stage and is believed to contribute to acrosome biogenesis by interacting with GOPC (Banu et al., 2002; Hicks and Machamer, 2005; Bentson et al., 2013). Another important protein that is involved in protein transport is protein interacting with C kinase 1 (PICK1), which is primarily localized around the Golgi apparatus in spermatids (Arvan and Castle, 2013). PICK1 regulates vesicle trafficking from the Golgi apparatus to the developing acrosome by interacting with GOPC (Xiao et al., 2009). Hence, Golgi-associated proteins are of great significance in vesicle formation and trafficking during acrosome biogenesis. Autophagic machinery also participates in acrosome biogenesis by regulating vesicular trafficking. Autophagy refers to the intracellular catabolic pathway which is responsible for the degradation and recycling of organelles and cytosolic proteins via autophagosomes (double-membrane vesicle) (Yang and Klionsky, 2009; Mizushima and Levine, 2010). Microtubule-associated protein 1A/1B-light chain 3 (LC3) activated by autophagy related protein 7 (ATG7) is delivered to Golgi-derived vacuoles either directly or indirectly (via phagophores) where the activated protein either facilitates the fusion of vesicles or guides them toward the nucleus (Wang et al., 2014). These investigations suggest that the autophagic pathway might be involved in acrosome biogenesis.

Proacrosomal granules undergo fusion with each other to form a single large acrosomal granule at the nuclear surface. Vesicle fusion requires SNARE complexes that help in the fusion of opposing membranes (Rizo and Südhof, 2002; Ungermann and Langosch, 2005; Zhao and Brunger, 2016). Moreover, the disruption of fatty acid desaturase 2 (FADS2) results in Syntaxin 2 scattering, which eventually impairs acrosome formation (Roqueta-Rivera et al., 2011). TATA element Modulatory Factor (TMF/ARA160) is another Golgi-associated protein required for the fusion of vesicles to the targeted membrane (Bel et al., 2012; Miller et al., 2013), and interruption of its expression leads to a complete absence of the acrosomes, suggesting that TMF/ARA160 probably supports the transport and docking of proacrosomal vesicles to the nucleus (Lerer-Goldshtein et al., 2010). Human Rev-binding (HRB) is another critical protein required for the docking of Golgi-derived proacrosomal vesicles; it binds to the cytosolic side of proacrosomal vesicles and links the Golgi apparatus and the nuclear surface (Kang-Decker et al., 2001). Polypeptide N-acetylgalactosaminyltransferase 3 (GALNT3) is located in the cis-medial region of the Golgi, and its disruption leads to failure of proacrosomal vesicles fusion and transport to nuclear surface suggesting the significance of protein O-glycosylation in acrosome biogenesis (Miyazaki et al., 2013). Besides, many other proteins important for vesicle formation or trafficking such as SMAP2 and GOPC also contribute to the fusion of proacrosomal vesicles (Yao et al., 2002; Funaki et al., 2013).

**Figure 3:**
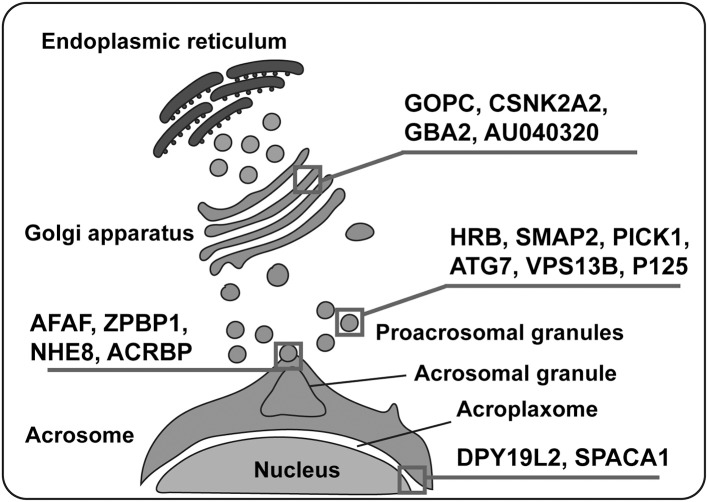
Various proteins are involved in acrosome biogenesis. Several proteins play significant roles in the whole process of acrosome biogenesis, which can be divided into four processes: vesicle formation, vesicular trafficking, vesicular fusion, and acrosome binding to the nucleus. Squares depict the main action of various proteins mentioned in the figure.

In addition to the transportation to the concave region of the nuclear surface and fusion to form a single large acrosomal granule, the attachment and spreading of the acrosome over the nucleus is also very important to its function. Sperm acrosome-associated 1 (SPACA1), an acrosomal membrane protein, participates in the process of acrosome attachment to the nucleus and the disruption of SPACA1 leads to the detachment of the acrosome from the nucleus (Fujihara et al., 2012). Zona pellucida-binding protein 1 (ZPBP1) is another important protein localized on the periphery of the acrosomal membrane (Lin et al., 2007; Yu et al., 2009), its absence results in the compaction failure of the acrosome and subsequently leads to acrosome fragmentation (Lin et al., 2007). Fer Testis (FerT) is a member of Fes/Fps (nonreceptor tyrosine kinases family), and it regulates cytoskeletal reorganization, cell adhesion, and vesicular transport (Greer, 2002) by attaching itself to the cytosolic surface of OAM and coexists with phosphorylated cortactin (an F-actin regulator protein) in the acroplaxome (Kierszenbaum, 2006; Kierszenbaum et al., 2008). Developmental pluripotency-associated 19-like 2 (DPY19L2) acts as a bridge between the nucleus and the acroplaxome, and its deficiency leads to a loss of the acrosome due to disruption of the nuclear/acroplaxome junction (Pierre et al., 2012). Similarly, disruption of Lamin A/C (a structural component of the nuclear envelope) (Dittmer and Misteli, 2011) leads to acrosome fragmentation and malformed acrosome (Shen et al., 2014). A vast array of specific proteins is involved in acrosome biogenesis and any defect may result in malformation of the acrosome and eventually lead to infertility (Table 1; Figure 3).

**Table 1:** Mouse models related to acrosome biogenesis.

	**Protein**	**Function**	**Phenotype**	**References**
Vesicle formation and trafficking	Hsp90b1	Folds, degrades, and activates ER proteins	Globozospermia	Audouard and Christians, 2011
	GBA2	Glycolipid hydrolase	Globozospermia Glucosylceramide accumulation Disruption of Sertoli-germ cell communication	Yildiz et al., 2006
	GOPC	Transport of proacrosomal granules from Golgi to the acrosome	Globozoospermia Absence of perinuclear theca Absence of coiled-coiltail	Ito et al., 2004; Suzuki-Toyota et al., 2004
	Hrb	Nucleocytoplasmic trafficking Proacrosomal vesicles fusion Acroplaxome formation	Globozoospermia Weak acrosomal vesicle binding to nucleus	Kang-Decker et al., 2001; Kierszenbaum et al., 2004
	Vps54	Endosomes to the trans-Golgi vesicular trafficking	Lack of acrosome Globozospermia	Paiardi et al., 2011
	Sirt1	Recruits LC3 to Golgi-derived vesicles Recruits GOPC and PICK1 to nucleus-associated acrosomal vesicles	Globozospermia	Liu et al., 2017
	Atg7	Recruits LC3 to Golgi-derived vesicles Recruits GOPC and PICK1 to nucleus-associated acrosomal vesicles	Globozospermia	Wang et al., 2014
	PICK1	Involved in proacrosomal granules trafficking	Globozospermia	Xiao et al., 2009
Vesicular fusion	GM130	Vesicular fusion into a single large acrosome vesicle	Lack of acrosomes Aberrant mitochondrial sheath assembly Globozospermia	Han et al., 2017
	AU040320	Vesicular fusion into a single large acrosome vesicle	Lack of acrosomes Globozospermia	Guidi et al., 2018
Anchor the acrosome to the nucleus	DPY19L2	Anchor the cytoskeleton to the nuclear envelope	Dissociation of acroplaxome Globozospermia	Pierre et al., 2012
	ZPBP1	Acrosomal protein	Globozospermia Acrosomal fragmentation Disassembly of protein matrix	Lin et al., 2007
	SPACA1	Supports sperm-egg interaction	Disrupted acroplaxome Failure of acrosome thinning	Fujihara et al., 2012
	Csnk2a2	Nucleus and acrosome formation	Lack of acrosome Acrosomal detachment from nucleus Acrosomal scrap	Xu et al., 1999
	SPAG4L/4L-2	Involved in nucleo-cytoskeleton complex formation Acrosomal vesicle docking to the nucleus	Globozospermia	Frohnert et al., 2010

Although most acrosomal substances are transported to the developing acrosome via the ER-Golgi route (Fawcett and Hollenberg, 1963; Clermont and Tang, 1985), numerous other routes are also suspected to exist for the transfer of acrosomal components to the developing acrosome. Toshimori (1998) has reported the existence of an extra-Golgi tract and a Golgi tract, which includes a Golgi-acrosomal granule tract and a Golgi-head cap tract (Toshimori, 1998).

## Potential Ways for the Origin of Acrosome

Previously, immunocytochemical investigations of glycoprotein synthesis in the Golgi established the acrosome as a direct Golgi derivate (Friend and Fawcett, 1974; Tang et al., 1982; Aguas and da Silva, 1985; Anakwe and Gerton, 1989; Moreno et al., 2000). Later, the acrosome was proposed to be a specialized lysosome based on an acidic pH, protease activities and the presence of hyaluronidase (Hartree and Srivastava, 1965; Allison and Hartree, 1970). A non-lysosomal origin of the acrosome has also been proposed (Martínez-Menárguez et al., 1996a). Martínez-Menárguez et al. (1996a) reported the absence of two well known lysosomal markers; lysosomal membrane glycoprotein (Igp) 120 and mouse Lamp-1 in acrosomal membranes. Moreover, acrosomal and proacrosomal vesicles both lacked two important endosomal markers, cation-dependent and -independent mannose 6-phosphate receptors suggesting some lysosomal features are absent in the acrosomes (Martínez-Menárguez et al., 1996a). In addition, small GTPases were found to be associated with acrosome development (Ramalho-Santos et al., 2001), leading researchers to postulate that the acrosome is a unique cellular organelle, and could be considered a secretory granule (Moreno, 2003). More recently, the acrosome has been suggested as a novel lysosome-related organelle (LRO) (Berruti et al., 2010; Berruti and Paiardi, 2011, 2015). The details of our understanding about acrosome biogenesis are given below:

### Is the Acrosome a Direct Golgi Derivative?

The release of acrosomal contents, during AR, led to concerns and questions about how acrosomal substances are synthesized and stored. To answer these questions, researchers turned their center of investigation toward biosynthetic pathways, especially those of the trans-Golgi network. In line with this, the synthesis, target, and fate of a number of acrosomal proteins in conjunction with lysosomal (Lamp-1, cathepsin D) and Golgi markers (giantin, β-COP, golgin 97) have been precisely examined using immunocytochemical techniques (Aguas and da Silva, 1985; Anakwe and Gerton, 1989; Martínez-Menárguez et al., 1996b; Moreno et al., 2000; Ramalho-Santos et al., 2001). Subsequently, some of these acrosomal-associated proteins were found to be produced in the Golgi complex of spermatocytes (cells that lack an acrosome) and later transported to the acrosome (Anakwe and Gerton, 1989; Escalier et al., 1991). These results were in complete accordance with the previous findings (Fawcett and Bloom, 1986). Therefore, the acrosome was interpreted to be directly derived from the Golgi complex, which acts as a source for membrane and protein contents. In other words, the TGN could be considered the main player during acrosome development, and no lysosome-related characteristics needed to be attributed to the acrosomal vacuole. Meanwhile, more investigations were carried out to identify the origin of acrosomal proteins, their trafficking, and sorting. Consequently, some novel roles of acrosomal proteins were revealed. For instance, acrosin was found to be stored in an inactive state as proacrosin and only activated by the protease acrolysin (McRorie et al., 1976) at the time of AR. Activated acrosin accelerates the release of acrosomal contents by dispersing the acrosomal matrix (Mao and Yang, 2013). Therefore, acrosin is now believed to help in not only the cleavage and subsequent activation of acrosome-specific proteases but also their release via exocytosis (Mao and Yang, 2013). These results were consistent with previous findings (Baba et al., 1994) that showed disruption of acrosin did not affect fecundity but led to a delayed fertilization rate in mice. However, some data exist that are not concordant with the idea that the acrosome is directly derived from Golgi. For instance, the Golgi apparatus detaches from the acrosomal space and moves in the opposite direction during the late capping phase. Furthermore, differentiating spermatids are characterized by the presence of an atypical micro-tubular organization. For example, a cortical microtubule array, despite the absence of centrosome, has been observed in the Golgi phase and tends to disappear during the formation of the manchette, which is a transient structure over the nuclear envelope at the late capping phase (Cherry and Hsu, 1984; Moreno and Schatten, 2000). In fact, this idea is so deep-rooted in the field that a variety of proteins in somatic cells that are involved in endocytosis have also been investigated, and were found to play a role in the biosynthetic/anterograde pathway, supporting acrosome biogenesis in spermatids (Berruti and Paiardi, 2011, 2015). Hence, in spite of contrary reports, a large majority of the reproductive-research community still supports the idea that the acrosome is a direct Golgi derivative.

### Is the Acrosome a Secretory Granule?

The notion that the acrosome is a direct Golgi derivative prevailed until the emergence of reports strengthened earlier evidence for the presence of a range of hydrolytic enzymes and a low pH maintained by the activity of V-ATPase (Sun-Wada et al., 2002). These characteristics were found to be common between lysosomes and acrosomes. Moreover, two Rab family members were identified to be involved in endocytosis and acrosome development, such as Rab5 (Simonsen et al., 1998) and Rab7 (Ramalho-Santos et al., 2001), respectively. Although acrosomes and lysosomes share several common characteristics, there are also dissimilarities. As mentioned earlier, the acrosome has been suggested to be a modified secretory granule (Moreno and Alvarado, 2006). For instance, serine proteinases (unique to testis), AM67 (a secretory component protein), acrosin acrogranin (Ohmura et al., 1999; Abou-Haila and Tulsiani, 2000) and exocytotic properties support the idea that the acrosome could be considered analogous to a secretory granule. Secretory granules are known to carry luminal protein contents that are directly delivered by the biosynthetic pathway to the targeted organelle and do not traverse to other parts of the endosomal system (Arvan and Castle, 1998). Secretory lysosomes, however, receive proteins through biosynthetic and endocytic pathways and serve as both degradative and secretory compartments (Blott and Griffiths, 2002). Secretory lysosomes are also mostly found in hematopoietic lineage-derived cells. To solve the mystery of whether the acrosome is really a secretory lysosome, Lamp-1 and Lamp-2 (lysosome specific proteins) were studied in detail during spermiogenesis. Results showed that both Lamp-1 and Lamp-2 were found to link with cytoplasmic vesicles only and not to the growing acrosome (Moreno, 2003). Although AR is usually associated with a somatic cell exocytosis, it still has several exclusive characteristics. For instance, AR is an irreversible “all-or-nothing” event. Moreover, in contrast to a single large vacuole of sperm, there are several secretory vesicles in cells that show exocytosis. In addition, AR takes place only once in each sperm because once the acrosome has reacted, it cannot be replaced by further biogenesis. Similarly, acrosomal membranes are lost and cannot be recycled at the time of AR (Berruti, 2016). In each sperm, only a single secretory granule exists, in contrast to the numerous secretory vesicles found in most other exocytotic cells. A feature that makes the acrosome unique is that the acrosome remains undocked prior to the required exocytosis stimulus, which is in contrast to other granules that are docked even before the application of the relevant stimulus (Table 2; Zanetti and Mayorga, 2009; Tsai et al., 2010; Rodríguez et al., 2011, 2012). Another interpretation has also been made in regards to secretory granule/lysosome nature of acrosome. Sperm traversing through the zona pellucida doesn't show any enzymatic lysis in eutherian (Bedford, 1998). A physical thrust, from the sperm head's structure was implicated in vitelline coat invasion (Bedford, 1998; Berruti, 2016). This investigation pointed out the importance of the “exocytotic” release of the acrosomal contents to penetrate the zona pellucida.

**Table 2:** Similarities and differences between acrosome and secretory granules of other exocytotic cells.

**Feature**	**Sperm (acrosome)**	**Exocytotic cells (secretory granule)**
**SIMILARITIES**		
Secretory components in common	AM67	AM67
Exocytosis	Present	Present
Enzymes in common	Serine proteinases	Serine proteinases
**DIFFERENCES**		
No. of secretory granules	Single (one)	Numerous
Granular docking	Undocked	Docked
Exocytosis	Once	Multiple times
Regeneration/replacement	Once reacted, can't be replaced	Can be replaced or reproduced
Membrane recycling	No membrane recycling	Membranes are recycled

### Is the Acrosome a Lysosome-Related Organelle?

The acrosome contains a vast array of acidic hydrolytic enzymes that are essential for the AR to take place normally and help the sperm to bind and dissolve the oocyte coverings during the exocytic release at the time of fertilization (Hartree and Srivastava, 1965; Allison and Hartree, 1970; Jin et al., 2011). Initially, researchers identified the lysosomal enzyme, hyaluronidase, and later several other hydrolytic enzymes such as acid phosphatase (another lysosomal enzyme), glycohydrolases, proteases, esterases, and aryl sulfatases in the acrosomes. These findings led to the suggestion that the acrosome is nothing unique, but a specially modified lysosome that has evolved to facilitate the fertilization process (Hartree and Srivastava, 1965; Allison and Hartree, 1970; Zaneveld and De Jonge, 1991; Tulsiani et al., 1998). Further strengthening this hypothesis was the characterization of analogous histochemical properties, the acidic pH of both the organelles (Allison and Hartree, 1970) and pro-acrosomal vesicle biogenesis in the Golgi apparatus (Table 3; Burgos and Fawcett, 1955; Dooher and Bennett, 1973). Among all the acrosomal enzymes, acrosin remains the most well-characterized protease (McRorie and Williams, 1974), but its location in the acrosome remains controversial. Is acrosin associated with the IAM, in the vicinity of the acrosomal membrane, or in the acrosomal matrix (Polakoski and Zaneveld, 1976; Shams-Borhan et al., 1979; Berruti and Martegani, 1982, 1984; Castellani-Ceresa et al., 1983)? In contrast, many believed acrosin existed to help sperm progress through the zona pellucida, and was therefore named the zona pellucida proteolytic enzyme/zona-penetrating enzyme (Chang and Hunter, 1975). Previously, a main research focus was to determine the function of different hydrolytic enzymes, especially acrosomal enzymes involved in the swift focal lysis of the outer coverings of the oocyte for fertilization. Researchers believed that the acrosome was a specialized lysosome, and enzymatic lysis would be required for sperm–oocyte fusion. But no consensus existed to unite both of those ideas (Bedford, 2014). The belief that the “acrosome is a specially modified lysosome” was finally shattered with the advent of gene-knockout technology, which revealed that acrosin is not indispensable for fertilization (Baba et al., 1994). In the past few years, the acrosome was suggested to be a lysosome-related organelle (LRO) based on findings that acrosome biogenesis involves both the biosynthetic and endocytic pathways (Berruti et al., 2010; Berruti and Paiardi, 2011).

**Table 3:** Similarities between acrosome and lysosome.

**Feature**	**Acrosome**	**Lysosome**
Enzymes in common	Hyaluronidase, proteinases (acrosin), esterases, neuraminidases, acid phosphatases	Hyaluronidase, proteinases, esterases, neuraminidases, acid phosphatases
pH	Acidic	Acidic
Origin	Golgi Apparatus	Golgi Apparatus

LROs are special membrane-bound organelles that received cargo from early endosomal intermediates and link biosynthetic and endosomal systems (Delevoye et al., 2009). LROs show a unique morphology, composition, and physiology and represent the resident cell. It has been suggested that the acrosome is an LRO, and it receives diverse protein cargos from more non-redundant pathways that contribute to acrosome biogenesis. It could therefore be construed that the lytic contents are primarily delivered from the ER-Golgi-TGN route while the acrosomal matrix scaffold and membranous constituents are contributed by the early endosome–endosome intermediates-TGN route. In addition, the complexity of the structure and physiology of the mature LRO is supported by the involvement of several routes. In line with this, several acrosomal features such as high spatial regulation of acrosome biogenesis, highly polarized location, and “modular” organization of its ingredients are consistent with the proposed nature of the acrosome as an LRO (Marks et al., 2013). Proteomic analysis data (Guyonnet et al., 2012) also uncovered unique analogies of biogenesis and protein contents between acrosomes and LROs. For instance, melanosome biogenesis follows four developmental stages (Seiji et al., 1963) that are similar to acrosome biogenesis. Furthermore, both the melanosome matrix and the acrosomal matrix can self-aggregate. Theses matrices both possess a core made up of a firm amyloidogenic structure that is later transformed into a functional matrix by successive attachment of several proteins (Delevoye et al., 2009; Guyonnet et al., 2012). Recently, we found that germ cell-specific *atg7*-knockout mice produce a globozoospermia-like phenotype due to a malformed acrosome, and autophagy was found to mediate the proacrosomal vesicle transport or fusion in the acrosome (Wang et al., 2014). Thus, the acrosome is proposed to have originated from an autolysosome rather than a lysosome alone. In support of this hypothesis, Sirt1 (sirtuin 1) and Tbc1d20 (TBC1 domain family, member 20) have been found to be involved in acrosome biogenesis and regulate autophagic flux (Sidjanin et al., 2016; Liu et al., 2017).

## Perspective and Concluding Remarks

Although the origin and the first appearance of acrosomes are yet to be determined, it could have originated from the simplest eukaryotes, such as yeast. A prerequisite for yeast mating is the formation of shmoo tips/ projections (Merlini et al., 2013), which show several features similar to the typical acrosome, such as the presence of degradative enzymes and the trafficking of some vesicles. Therefore, the primary form of the acrosome could have developed very early in the evolutionary history. Nevertheless, the existence of a true acrosome can be traced back to the evolution of heterogamy, heterozygosity, and cross-fertilization. Initially, all organisms were homogametic, and most were self-fertilizing. Later, some organisms evolved cross-fertilization. To better adapt the ever-changing circumstances of the earth (Shields, 1982; Schmidt-Rhaesa, 2007; Stearns, 2013), protective coverings around eggs developed to ensure gamete integrity, and sperms needed to arm with a powerful weapon in its arsenal, the acrosome, to evade these protective gamete vestments (Schmidt-Rhaesa, 2007). This review provides a brief historical overview and highlights new break-through on acrosome biogenesis. The acrosome might be generated from a combination of many membrane trafficking systems during gametes fusion, and it might have evolved during the arms race between sperm and ovum. A collective effort to uncover unidentified components, their interactions, and their regulatory mechanism(s) is urgently needed to elucidate a more complete picture of this highly complicated secretory vesicle. Our current era definitely will be a time of deep understanding of acrosome biogenesis.

## Author Contributions

MK collected the data, drew the figures, and wrote the manuscript. HG revised the figures and the manuscript. WL proposed the idea and revised the manuscript.

### Conflict of Interest Statement

The authors declare that the research was conducted in the absence of any commercial or financial relationships that could be construed as a potential conflict of interest.
